# Assessment of treatment plans from high-dose-rate-brachytherapy of prostate cancer in Nigeria: findings from pioneer centre

**DOI:** 10.3332/ecancer.2025.1906

**Published:** 2025-05-15

**Authors:** Bidemi I Akinlade, Iyobosa B Uwadiae, Abbas A Abdus-Salam, Atara I Ntekim, Ayorinde M Folasire, Mutiu A Jimoh, Afolabi A Oladeji, Foluke O Sarimiye, Adeniyi A Adenipekun

**Affiliations:** 1 Department of Radiation Oncology, Faculty of Clinical Sciences, College of Medicine, University College Hospital, University of Ibadan, Ibadan 200212, Oyo State, Nigeria; 2 Department of Radiation Oncology, University College Hospital, Ibadan 200212, Oyo State, Nigeria

**Keywords:** high dose rate brachytherapy, prostate cancer, temporary prostate implant, treatment plan quality, dose optimisation, organs at risk, dose volume parameters

## Abstract

**Introduction:**

High dose rate (HDR) brachytherapy is a promising therapeutic approach for localised prostate cancer. Optimised treatment plans have been shown to improve disease control and reduce toxicity on the organs-at-risk (OAR).

**Aim:**

To report findings from the treatment plan parameters (TPPs) obtained from two different HDR brachytherapy treatment regimens at the University College Hospital, Ibadan Nigeria.

**Methods:**

The treatment plans of 90 patients, who had HDR brachytherapy to the prostate gland between August 2020 and October 2023 were considered. All were treated with Bebig Saginova machine, housing Cobalt-60 source, the first multi-channel unit in the country then. 44% of patients received dose of 18 Gy in 2 fractions (category A), while the remaining received 27 Gy in 2 fractions (category B). Treatment plans were generated on the Sagiplan 2.0 treatment planning system from Eckert & Ziegler, BEBIG and relevant TPP were extracted and analysed using IBM SPSS 27.

**Results:**

The mean age(years) of patients in Categories A and B were 65.3 ± 6.59 and 66.5 ± 5.32, respectively; their gleason score and prostate-specific-antigen were (7 ± 1; and 7 ± 1) and (12.83 ± 16.32; and 12 ± 17 ng/mL), respectively. The mean volume (cm^3^) of prostate volume (PVol.) for both categories were 46 ± 21 and 31 ± 8, respectively. The paired *t*-test performed on TPP from patients in both categories was statistically significant (*p* < 0.005), except their age (*p* < 0.873) and dose homogeneity index (*p* < 0.639). Also, the regression analysis showed that V_100_ is statistically dependent (*p* < 0.05) on Total Reference Air Kerma, conformal index, Rectum D_10_ and PVol. in both categories.

**Conclusion:**

Although, some level of optimal dose coverage around the prostate gland was achieved for some of the patients, especially those in Category B, there is still room for improvement to minimise the dose to OAR.

## Introduction

High dose rate (HDR) brachytherapy is a form of radiotherapy where radioactive sources are directly implanted into the target to achieve a highly conformal therapy. It is an effective method of treating localised prostate cancer which is the most common cancer among men of age 50 years and above. Low dose rate (LDR) permanent seed brachytherapy is most commonly used for low-risk cancer, while HDR is usually combined with external beam radiotherapy (EBRT) to treat higher risk disease [1]. The HDR of radiation dose deliverable and the possibility of the large fractional size of radiation employed in HDR techniques have been found to be a radiobiological advantage, especially for tumours with high sensitivity to radiation fractional size and can be administered as a monotherapy or as a boost with adjuvant EBRT [2, 3].

Effective treatment of prostate cancer requires a good radiation dose distribution around the target volume and minimal dose to the surrounding healthy tissues [4]. In HDR, treatment is usually delivered using a high-strength stepping source with treatment planning and dwell time optimisation performed after treatment applicators (plastic or steel needles) have been implanted [5]. This assures dosimetry that is more consistent when compared with LDR brachytherapy permanent seed implants which usually faces challenges of seed misplacement, seed displacement and swelling of the prostate. All of these may result in variations between the planned and delivered doses [6]. The quality of treatment plan parameters for prostate cancer depends largely on the operator’s skills, the prostate gland anatomy, the prostate volume (PVol) and the surrounding tissues (organs-at-risk (OAR)) among others [7]. As HDR continues to evolve, a strong focus on treatment plan quality (TPQ) is expected to further enhance its effectiveness in managing localised prostate cancer.

Our centre is the first radiotherapy centre in Nigeria to acquire a full-fledged multi-channel afterloading HDR facility which is required to treat patients living with prostate cancer using a temporary implant. This study presents our initial findings with respect to dosimetry procedures: dose volume parameters (DVPs) and TPQ, obtained from the treatment planning system of 90 patients managed between August 2020 and October 2023.

## Materials and methods

Ninety patients living with prostate cancer and managed in the Department of Radiation Oncology, University College Hospital (UCH), the pioneer centre to offer HDR temporary prostate brachytherapy in Nigeria, between August 2020 and October 2023 were considered in this study. Forty patients received a prescription boost dose of 18 Gy in 2 fractions (9 Gy per day) to complement EBRT of 45–50 Gy, while 50 patients received a monotherapy dose of 27 Gy in 2 fractions (13.5 Gy per day). All fractions were completed within 1 week of each patient’s admission to the ward.

The patients were carefully selected to ensure that they were in the early stages of the disease limited to the prostate. Most of the patients (64%) were in TNM stages 1 and 2 and more than 70% had prostate-specific antigen (PSA) below 20 ng/mL. Only 12% had TNM stage 4 diseases while 24.6% had PSA above 20 ng/mL. None of the patients had distant metastases to the lungs, liver, bones or the nervous system at the time of treatment.

All patients were treated with the Bebig multi-channel remote afterloading HDR brachytherapy unit, model SagiNova. This machine was manufactured by Eckert & Ziegler Bebig GmbH, Berlin Germany. It was acquired and installed between 2018 and 2019 through a public–private partnership initiative between UCH and a private investor. The HDR unit uses a radioactive Cobalt-60 source with a half-life of 5.26 years. The source strength as at the time of installation (16 October 2018) was 23.43 mGym^2^/h. It has the capacity to deliver a nominal dose rate of 12 Gy/hr or higher at the prescription point. This type of radioactive source greatly relieved the burden of quarterly source replacements, which are usually associated with challenges of foreign exchange, customs clearance, shipping and transportation among others.

The image sequences used for treatment planning of all the patients were obtained from an Ultrasound machine, Flex Focus 400 from bK Medical, Germany, after the insertion of appropriate number of needles, first at the peripheral and then at the internal, while observing the spacing distance of at least 5 mm between needles. These images are then used by the treatment planning software from Bebig, SagiPlan 2.0, for planning the treatment. The treatment planning procedures include calibration or matching of the physical prostate template with the electronic template, contouring of the treatment volume (Prostate gland) and OAR (Urethra and Rectum). Contouring of the bladder is not required when using Ultrasound imaging technique.

All patients underwent standard HDR brachytherapy treatment procedures/protocols which were adopted from the GEC/ESTRO recommendations [8]. The patients’ selections and other procedures before actual treatment were as earlier reported by Abdus-Salam *et al* [9] in our first publication on prostate HDR brachytherapy experience.

Treatments were delivered using a high-strength stepping source (Cobalt-60) with planning and dwell time optimisation (inverse planning) performed after the needles had been properly implanted under ultrasound guidance and carefully identified on the images in the planning software [10, 11]. The planning software have the capability to calculate dose distribution per volume of the organ, based on the parameters set as constraints (dose volume histogram) and determine the overall plan quality. These are usually arrived at after several mathematical iterations, dose optimisation/adjustment of the isodose curves around the contoured organs and then display for assessment and approval. The patients are usually treated after these treatment plans are approved by the Radiation Oncologists. For the purpose of this study, the following was extracted from the approved treatment plans: conformal index (COIN), dose homogeneity index (DHI), Total Reference Air Kerma (TRAK) and total treatment time (min) used to deliver the prescribed fractional dose. TRAK is the total radiation exposure when the source is outside of its shielded position. While DHI is the tool used to analyse the uniformity of dose distributions in the target volume, COIN represents the relationship between isodose distributions and the target volume coverage. Other parameters documented for this study are the patient’s age, Gleason score (GS), PSA (GS and PSA are values before the HDR brachytherapy procedure), number of inserted needles (peripheral and internal), DVP of the contoured organs’ of interest namely: Prostate (Dose received by its minimum volume (D_min_), dose received by 90% of its volume (D_90_), the volume that received 100% of the prescribed dose (V_100)_ and the volume that received 150% of the prescribed dose (V_150_)); Urethra (Dose received by 10% of its volume (D_10_) and the maximum dose received (D_max_)); Rectum (Dose received by 10% of its volume (D_10_) and the maximum dose received (D_max_)). Bladder is not quantified among OAR for ultrasound-planned dosimetry [12].

These data were analysed using IBM SPSS statistics 27, and the results are presented in Tables 1–4. Part of the analysis was to compare the mean values of all the variables obtained for patients in category A with those in category B using a paired samples *T*-test and to determine the variables that have statistical significance (*p* < 0.05) on the volume of the prostate that will receive 100% of the prescribed dose (V_100_). To achieve the latter, V_100_ was set as the dependent variable, while the Rectum D_max_, Rectum D_10_, Urethra D_max_, Urethra D_10_, PVol contoured, COIN and TRAK were set as predictor variables.

## Results

Out of the 90 patients managed with the remote afterloading HDR unit for interstitial temporary prostate implants, 44% received a total dose of 18 Gy in 2 fractions (category A), while the remaining 56% received a total dose of 27 Gy in 2 fractions (category B). All patients had one fraction per day and completed their treatment within a week. The patients’ prognostic factors such as GS and PSA values before the treatment and the age of the patients are presented in Table 1 for those in categories A and B. The mean age of patients in category A (65.3 ± 6.59) and category B (66.5 ± 5.32) were almost the same (*p* < 0.873) and so were their GS and PSA values (*p* = 0.000). Also presented in Table 1 is the average volume (cm^3^) of the prostate gland treated for patients in category A which was significantly larger (46 ± 21) than those in category B (31 ± 8) (*p* < 0.001). The number of implanted needles used for treatment of patients in category A were smaller (13 ± 2) than those in category B (16 ± 3), (*p* < 0.001). Also presented in Table 1 for patients in categories A and B are DVP in percentages ((median (range)) of the prescribed dose and volumes for the prostate and OAR (urethra and rectum).

The summary of the regression analysis performed on the DVP and the TPQ to determine their relative dependence or statistical significance on V_100_ at the 95% confidence interval is presented in Tables 2–4.

## Discussion

As the pioneer Oncology centre for interstitial temporary prostate implant with HDR in Nigeria, the TPQ and DVP obtained from the treatment planning of 90 patients living with prostate cancer have been assessed and analysed. The age of the patients considered in this study ranged from 51 to 78 years. This is consistent with the age at which the incidence of prostate cancer is high (1 in 52 males) and hence the recommendation that screening should take place at age 50 years for men who are at an average risk of prostate cancer [13].

The patients were further divided into two categories: those treated at the commencement of the HDR clinical services (Category A) and those treated after modification of the treatment regimen (Category B). This modification followed some months of clinical observation of the treatment outcomes of the previous regimen. The patients in category A (44%) received a total dose of 18 Gy in 2 fractions as an adjuvant therapy to EBRT [14] while those in category B (56%) received 27 Gy in 2 fractions as monotherapy. These dose prescriptions were similar to those reported in literature, when HDR is given as a monotherapy [2, 15, 16]. It has also been reported that the use of HDR as monotherapy technique seems to be a promising treatment modality for low-risk prostate cancer [5]. The mean volumes of the prostate glands contoured for patients in categories A and B were within the recommended volume (≤ 60 cm^3^) of the prostate gland that should be implanted for HDR [4, 8, 16]. Also, the mean number of needles used for the prostate implants for patients in categories A and B were similar to the number of needles inserted for patients in the study reported by Kini *et al* [15]. In another study by Adam *et al* [7], they suggested that the minimum number of needles implanted for prostate cancer HDR should be greater than 9 and optimally greater than or equal to 13.

The DVP considered in this study (for prostate: D_min_, D_90_, V_100_ and V_150;_ for urethra: D_10_ and D_max;_ and for rectum: D_10_ and D_max_) for patients in both categories were among the parameters recommended for reporting for HDR prostate implants by GEC/ESTRO [8]. Their respective values obtained for patients in both categories were statistically significant (*p* < 0.001). The median values of DVP obtained were within the range of values reported in the literature [7] namely prostate D_90_ (65.9%–102.8%); urethra D_10_ (78.8%–152.9%); rectal D_10_ (37.4%–101.0%). The median volume of the prostate tissue covered by the 100% isodose surface (V_100_) in category A (64.20%) and category B (65.40%) were lower than the range of values (75%–99%) reported in the literature [14]. Likewise, the minimum doses to the target volume which was 40.67% of the prescribed dose in category A and 42.37% in category B were lower than the range of values reported (45%–97%) by the same authors. The median prostate D_90_’s obtained for category A (66.0%) and category B (73.96%) were lower than the value (110%) reported in literature [17]. However, the median urethra D_10_’s and rectum D_10_’s obtained in this study for category A (134.17%, 57.72%) and category B (150.85%, 60.52%), respectively, were higher than the values (114%, 55%) reported by the same authors.

Similarly, the Plan Quality Parameters (COIN, DHI, TRAK and total treatment time) obtained and compared between the two categories showed some level of significance (*p* < 0.001) in predicting V_100_ except for DHI (*p* < 0.639). The conformity of the isodose curve with the volume of the prostate gland (COIN) and homogenous distribution of dose (DHI) around the target volume obtained for patients in category A (39%, 56%) and category B (58%, 63%), respectively, were lower than the values (80%, 67%) reported by Major *et al* [17]. The TRAK, total treatment time, and the average number of needles obtained for patients in category A (3.26 mGy, 11.76 minutes and 13) were lower than those in category B (3.93 mGy, 16.53 minutes and 16), respectively. This variation may be due to the increases in the prescribed dose (18 Gy versus 27 Gy) and the number of treatment needles (13 versus 16) used for patients in category B. It is important to note that the greater number of implanted needles and increased PVol (PVol.) with improved D_90_ have been shown to produce better target coverage (V_100_), better dose distribution and decreased dose to the urethra (urethra D_10_) [7]. The regression analysis performed on some of the variables (COIN, TRAK, PVol., rectum D_10_, rectum D_max_, urethra D_10_, urethra D_max_) considered in this study was done to determine their statistical significance (*p* < 0.05) on V_100_ and showed (*R*^2^ = 0.995; direct relationship) that when all the variables are combined, they contributed significantly (*p* = 0.000) to produce better target coverage. However, on an individual basis, only COIN (*p* = 0.004), TRAK (*p* = 0.000), PVol. (*p* = 0.001), rectum D_10_ (*p* = 0.000) and number of needles (*p* = 0.001) have significance predicting V_100_.

## Conclusion

It can be seen from this study that if all the variables mentioned in this study are carefully acquired during treatment planning procedures, the resultant effects will be better coverage of the prostate gland and minimal dose to the OAR. Further study to assess the overall treatment outcome of these patients is still ongoing.

## Conflicts of interest

The authors have no conflicts of interest and therefore nothing to disclose.

## Funding

No funding was received.

## Figures and Tables

**Table 1.** Comparison between DVP and plan quality for patients in category A and B.

Parameters	Category A: 12 Gy in 2 fractions	Category B: 27 Gy in 2 fractions	*p*-value
Number of patients Average age (years) Average GS Average PSA (ng/mL) Average volume (cm^3^) Average No. of needles (Steel) Dose volume parameters (Median (Range)): Prostate D_min_ (%) D_90_ (%) V_100_ (%) V_150_ (%) Urethra D_10_ (%) D_max_ (%) Rectum D_10_ (%) D_max_ (%) Plan quality parameters (Median (Range)): COIN (%) DHI (%) TRAK (mGy) Total treatment time (min)	40 (44%) 65.3 ± 6.59 7 ± 1 12.83 ± 16.32 46 ± 21 13 ± 2 40.67 (14.44–74.11) 66.00 (51.00–78.00) 64.20 (26.30–90.30) 20.70 (3.90–70.60) 134.17 (103.89–209.22) 163.94 (108.89–440.78) 57.72 (23.67–90.89) 93.06 (30.22–160.89) 39.0 (15.0–67.4) 56.0 (38.0–69.0) 3.26 (1.41–6.01) 11.76 (5.05–21.35)	50 (56%) 66.5 ± 5.32 7 ± 1 12 ± 17 31 ± 8 16 ± 3 42.37 (17.78–56.22) 73.96 (32.67–89.48) 65.40 (34.50–80.00) 25.05 (13.90–34.90) 150.85 (113.78–181.56) 226.30 (138.96–369.26 60.52 (32.81–70.59) 93.44 (66.15–122.07) 58.0 (24.0–70.0) 63.0 (20.0–81.0) 3.93 (2.10–4.80) 16.53 (8.49–20.37)	0.873 0.000 0.000 0.001* 0.001* 0.001* 0.001* 0.001* 0.001* 0.001* 0.001* 0.001* 0.001* 0.001* 0.639 0.001* 0.001*

Paired *t*-test (2-tailed) * Statistically Significant

**Table 2.** Regression analysis between the DVP, plan quality parameters (Predictors) and V100 (Dependent variable).

Variables	Parameters
Predictors	Max. Dose to the Rectum, Rectum D10, Max Dose to the Urethra, Urethra D10, PVol., COIN, TRAK, number of needles
Dependent	Prostate Vol. that received 100% of prescribed dose (V_100_)
*R*-value	0.998
*R*^2^-value	0.995

**Table 3.** Significance of the predictors (combined) on the dependent variable.

ANOVA^a^						
Model		Sum of squares	df	Mean square	*F*	Sig.
1	Regression	3,115.778	7	445.111	626.254	0.000^b^
	Residual	15.637	22	0.711		
	Total	3,131.415	29			

a Dependent Variable: Prostate Vol. that received 100% Dose

b Predictors: (Constant), Max. Dose to the Rectum, rectum D10, Max Dose to the Urethra, COIN, PVol, urethra D10, TRAK, Number of needles

**Table 4.** Significance of individual predictor on the dependent variable.

Parameters		Unstandardized coefficients		Standardized coefficients		p-value
		B	Std. Error	Beta		
1	(Constant)	5.647	6.474		0.872	0.392
	COIN	34.416	10.848	0.430	3.173	0.004*
	TRAK	−12.070	1.681	−0.996	−7.180	0.000*
	PVol	0.568	0.140	0.452	4.043	0.001*
	Rectum D10	0.908	0.122	0.852	7.461	0.000
	Urethra D10	0.047	0.088	0.073	0.528	0.603
	Max dose to the urethra	0.017	0.015	0.107	1.167	0.256
	Max. dose to the rectum	0.039	0.081	0.064	0.481	0.635
	Number of needles	1.722	0.420	0.545	4.097	0.001*

*Paired *t*-test (2-tailed) * Statistically Significant
